# Transformation of Construction Cement to a Self-Healing Hybrid Binder

**DOI:** 10.3390/ijms20122948

**Published:** 2019-06-17

**Authors:** Werner E.G. Müller, Emad Tolba, Shunfeng Wang, Qiang Li, Meik Neufurth, Maximilian Ackermann, Rafael Muñoz-Espí, Heinz C. Schröder, Xiaohong Wang

**Affiliations:** 1 ERC Advanced Investigator Grant Research Group at the Institute for Physiological Chemistry, University Medical Center of the Johannes Gutenberg University, Duesbergweg 6, 55,128 Mainz, Germany; emad_nrc@yahoo.com (E.T.); shunwang@uni-mainz.de (S.W.); mneufurt@uni-mainz.de (M.N.); hschroed@uni-mainz.de (H.C.S.); 2 Polymers and Pigments Department, National Research Centre, Dokki, Giza 12622, Egypt; 3 Key Laboratory of Karst Dynamics, MLR & GZAR, Institute of Karst Geology, Chinese Academy of Geological Sciences, Guilin 541004, China; liqiang@karst.ac.cn; 4 Institute of Functional and Clinical Anatomy, University Medical Center of the Johannes Gutenberg University, Johann Joachim Becher Weg 13, 55,099 Mainz, Germany; maximilian.ackermann@uni-mainz.de; 5 Institute of Materials Science (ICMUV), Universitat de València, C/Catedràtic José Beltrán 2, Paterna, 46980 València, Spain; rafael.munoz@uv.es; 6 NanotecMARIN GmbH, Mühlstr. 19, 55,218 Ingelheim am Rhein, Germany

**Keywords:** Portland cement, inorganic polyphosphate, microcapsules, coacervate, concrete, microcracks, self-healing, soil bacteria

## Abstract

A new biomimetic strategy to im prove the self-healing properties of Portland cement is presented that is based on the application of the biogenic inorganic polymer polyphosphate (polyP), which is used as a cement admixture. The data show that synthetic linear polyp, with an average chain length of 40, as well as natural long-chain polyP isolated from soil bacteria, has the ability to support self-healing of this construction material. Furthermore, polyP, used as a water-soluble Na-salt, is subject to Na^+^/Ca^2+^ exchange by the Ca^2+^ from the cement, resulting in the formation of a water-rich coacervate when added to the cement surface, especially to the surface of bacteria-containing cement/concrete samples. The addition of polyP in low concentrations (<1% on weight basis for the solids) not only accelerated the hardening of cement/concrete but also the healing of microcracks present in the material. The results suggest that long-chain polyP is a promising additive that increases the self-healing capacity of cement by mimicking a bacteria-mediated natural mechanism.

## 1. Introduction

Portland cement, e.g., CEM I 42.5 R, is a widely used material that when mixed with water binds together to form concrete aggregates—small, stone-like pieces [1]. After addition of water to dry Portland cement powder, the material reacts under formation of calcium hydroxide, calcium silicate hydrate gel (C-S-H), and other hydration products. During this process, CO_2_ is taken up from air and initiates the carbonation process. Concrete, composed of fine to coarse aggregates that are bonded together with the cement paste has, in comparison to other composite materials, intrinsic micro-reservoirs that have the potency of self-healing [2]. In Germany, this inventive material was introduced 2000 years ago during the Roman time. While the Porta Nigra, a Roman gate in Trier, was built starting in 170AD in grey sandstone without using cement, soon after in the same city cement was used as dominant material for several structures in the Basilica of Constantine, which were built during the reign of the emperor Constantine I at the beginning of the 4th century. Interestingly, the interconnecting cavities between the stones in these structures show traces of self-healed microcracks, reflecting re-crystallization of calcite (to be published). In general, the materials that trigger the self-healing process in concrete have been grouped into two categories: first, autogenic healing materials; and second, autonomic healing materials [3]. Autogenic healing is initiated by endogenous, intrinsic materials within the cement, which have not yet reacted. In contrast, autonomic healing is caused by components that are normally not present in cement. In recent studies, progress has been achieved to implement self-healing property in the cement starting material, e.g., by addition of encapsulated liquid SiO_2_ precursors, sodium silicate, and MgO powder as healing materials [4,5]. Finally, increasing the crystal-growth seeds was found to contribute to self-healing potency [6]. The formation of cracks within concrete structures resulting in a disintegration of the engineered material is a worrying topic of increasing significance. It has been estimated that in the United States alone, repair and rebuilding costs of concrete infrastructure will be in the trillions of dollars [7]. In turn, the development of new binders and new cement with greater durability is urgently needed. As an example, the alkali-activated binders are mentioned, which emerged as an improvement to the ordinary Portland cement-based concrete [8].

In this study, we focus on a further improvement of Portland cement and its potency of self-healing. One major cause of microcrack formation is the process of drying shrinkage, which develops following cement hydration when moisture is driven out from the sample. During the drying process, a negative pressure develops inside the matrix pores as the result of the removal of water required for cement hydration [9]. The reduction of the mechanical strength is in parallel with crack formation [10]. Those cracks are formed not only as the result of the tensile stresses parallel to the drying surface, but also in response to the external restraints caused by the surrounding structures, such as the aggregates and reinforcements [9]. A further and severe cause for the introduction of cracks into cement is a sudden temperature shift, exposing the cement to a thermal gradient and thermal shock [11,12]. Under these conditions the material undergoes thermal fatigue followed by the introduction of locally formed cracks.

Consequently, intensive efforts have recently been undertaken to develop suitable expansive mineral additives [13,14,15]. A successful approach with a “dynamic” ingredient added to the cement was achieved by incorporating reactive MgO [16].

As was recently reviewed [17], independent cracks in cement can be sealed by bacteria-induced carbonate precipitation. This induced self-healing process can be initiated using the encapsulated bio-agents; the capsules can survive concrete mixing and are able to effectively reseal the cracks and simultaneously retain the mechanical properties and durability of the concrete material. The bacteria can be sustainably encapsulated and immobilized in Biochar [18].

Within the cement, and especially on its surface, Ca(OH)_2_ under consumption of CO_2_ is converted to CaCO_3_ and H_2_O [19]. This reaction from soluble Ca(OH)_2_ leaching out from the cement into the crack to solid CaCO_3_ is accelerated by bacteria present in the cement, which supply metabolic Ca^2+^ ions from their nutrients, such as calcium propionate Ca(C_3_H_5_CO_2_)_2_. This metabolite triggers CaCO_3_ formation under consumption of 7 moles of O_2_ and release of 5 moles of CO_2_ and 5 moles H_2_O. In this reaction, CaCO_3_ is formed directly in parallel to the autogenous healing. The second driving reaction following cement hydration is the formation of tobermorite (3CaO•2SiO_2_•3H_2_O) gel [20]. This phase controls bonding and filling properties of the cement during hydration and post-hydration.

Here, we introduce the concept of using polyphosphate (polyP) as an additive to cement. The bio-inorganic polymer, polyP, is an abundant component of all bacteria [21,22]. Linear polyp has been tested previously with chain lengths of 3 phosphate units or circular polyP with 6 units [23], and it showed a retarding effect on cement hydration. Instead of this, we use linear long-chain polyP with an average chain length of 40 phosphate units because of the following prominent characteristics of this polymer [24]. Firstly, long-chain polyP is a rich inorganic component in any bacteria in larger amounts [25], and in turn, the polymer is introduced into the constructing material via certain carbonate precipitating bacteria species [26]. This fact implies that bacteria may contribute to the self-healing process by supplying this polymer, polyP, to the damaged regions within the cement. Secondly, bacteria are known to hydrolyze polyP enzymatically by means of the alkaline phosphatase [27,28]. Consequently, ortho-phosphate produced from polyP supplied together with the cement or via the cement-associated bacteria will participate in the self-healing process. Thirdly, the material that is proposed in the present study to be added to the cement is the Na^+^ salt of the inorganic polyanion, polyP_n_*^n^^−^* (*n* = number of phosphate residues; Na-polyP). Consequently, in the presence of Ca^2+^ ions, the water-soluble Na-polyP is readily transformed into the only slightly soluble Ca-polyP, which, at a surplus of Ca^2+^ ions, forms amorphous Ca-polyP microparticles [29]. In cement, as in the study presented, its ingredient Ca(OH)_2_ will drive the reaction towards Ca-polyP formation and via the bacteria to crystalline Ca-phosphate. Fourthly, at more neutral pH conditions Na-polyP will undergo coacervate formation with Ca^2+^ prior to the transition to the solid Ca-polyP state [30]. Such a coacervate will allow the fabrication of a flexible and tightly fitting cement binder. Finally, the application of polyP as a binder appears to be commercially usable, since this polymer has a relatively low price and is needed only in small quantities. In the initial stage, after addition of polyP to the cement, in contact with water, polyP is stable and forms nano-microparticles [29].

With reference to the outlined suppositions, we describe that addition of Na-polyP not only accelerates the hardening process of the cement/concrete but also allows a fast release of mineralic salts to the microcracks, followed by a comparably fast self-healing process.

## 2. Results

### 2.1. Preparation of the Cement Samples

The details of the preparation of the respective cement samples, “CEM”–“hCEM”–“hCEM•polyP”–“Ca-polyP-MP”–“hCEM•Ca-polyP-MP”–“Ca-polyP-Emu” and “hCEM•Ca-polyP-Emu” are given under Materials and Methods in Section 4.2, Section 4.3 and Section 4.4.

**Figure 1:**
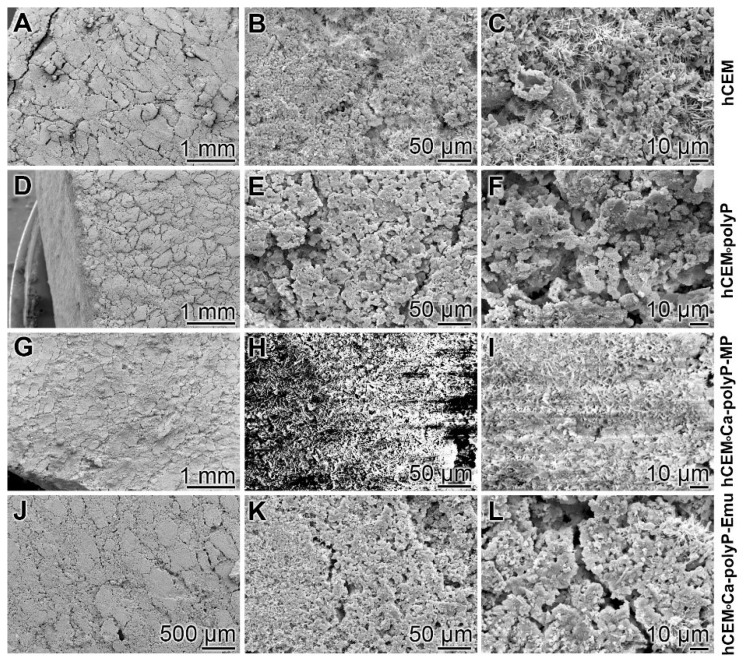
Surface structures of the different hydrated cement paste samples used, analyzed by SEM (scanning electron microscopy). (**A**–**C**) Hydrated control cement sample “hCEM”; (**D**–**F**) Na-polyP supplemented cement “hCEM•polyP”; (**G**–**I**) “Ca-polyP-MP” enriched cement “hCEM•Ca-polyP-MP”; and (**J**–**L**) “Ca-polyP-Emu”-containing cement “hCEM•Ca-polyP-Emu”. All samples were inspected without sputtering; under those conditions the “hCEM•Ca-polyP-MP” sample showed backscattering.

Microscopic analysis: The surfaces of the different hydrated cement paste samples were inspected by SEM (scanning electron microscopy) (Figure 1). The control sample “hCEM” (Figure 1A–C) shows the characteristic morphology of the hydrated cement paste [31]. Several crystalline structures can be discerned, among them the flaky, flat-jagged calcium silicate hydrate (C-S-H) crystals, hexagonally shaped calcium hydroxide, and ettringite needles (Figure 1C). All measure sizes were up to 10 µm. The surface of the “hCEM•polyP” is less structured and comprises an almost homogeneous layer of ≈1–2.5 µm microparticles (Figure 1D–F). The samples supplemented both with the “Ca-polyP-MP” and “Ca-polyP-Emu” particles and spheres “hCEM•Ca-polyP-MP” (Figure 1G–I) and “hCEM•Ca-polyP-Emu” (Figure 1J–L) show on their surfaces spherical micro- and -ultrafine particles of sizes of ≈1 µm (Figure 1I) and ≈3 µm (Figure 1L), respectively.

**Figure 2:**
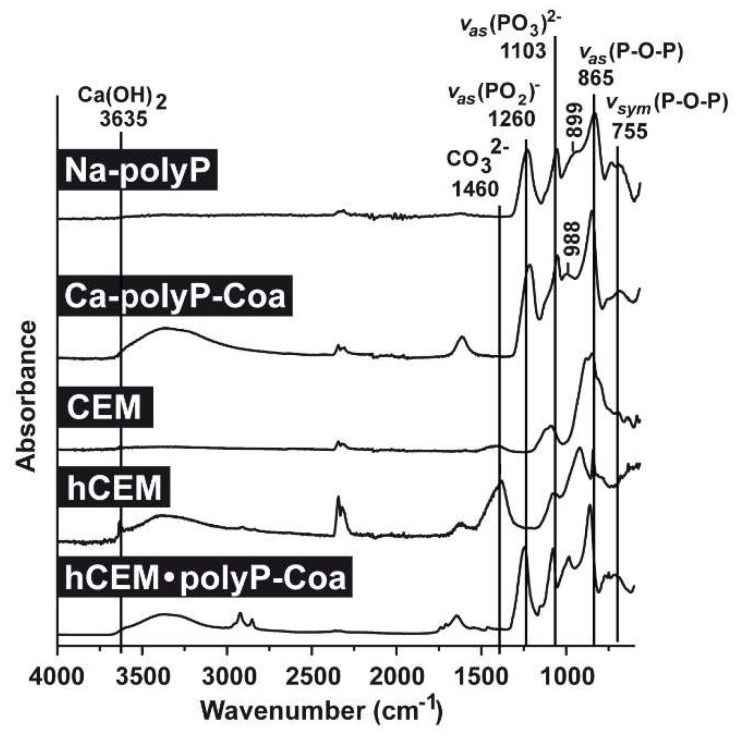
FTIR (Fourier-transform infrared spectroscopy) spectra of the different cement samples, “CEM” and “hCEM”, in comparison to the respective reference polyP samples, “Na-polyP” and “Ca-polyP-Coa”. “CEM” refers to the dry cement starting material, “hCEM” is the water mixed cement paste, and the coacervate formed onto “hCEM” is labeled “hCEM•polyP-Coa”. Both the polyP signatures and the characteristic cement vibrations are labeled.

FTIR analyses (Fourier-transform infrared spectroscopy): Comparative FTIR analyses were performed for the polyp-containing samples, with soluble Na-polyP and Ca-polyP coacervate as references, as well as the cement samples, the unhydrated cement “CEM”, the hydrated cement “hCEM”, and the coacervate that was formed onto “hCEM” after spreading of Na-polyP (Figure 2). The characteristic signatures for polyP are seen in the samples containing this polymer, such as the asymmetric (≈865 cm^−1^) and the symmetric (≈755 cm^−1^) vibrations, as well as the typical asymmetric stretching of the bridging (PO_2_)^−^ at ≈1260 cm^−1^. In addition, the asymmetric (≈1103 cm^−1^), the symmetric (≈899 cm^−1^), as well as the symmetric (≈988 cm^−1^) vibrations were recorded, which are characteristic for phosphate salts. Typical for cement are the signals recorded at 3635 cm^−1^ reflecting the (O-H) stretching of Ca(OH)_2_ and for carbonate at ≈1460 cm^−1^ (Figure 2).

**Figure 3:**
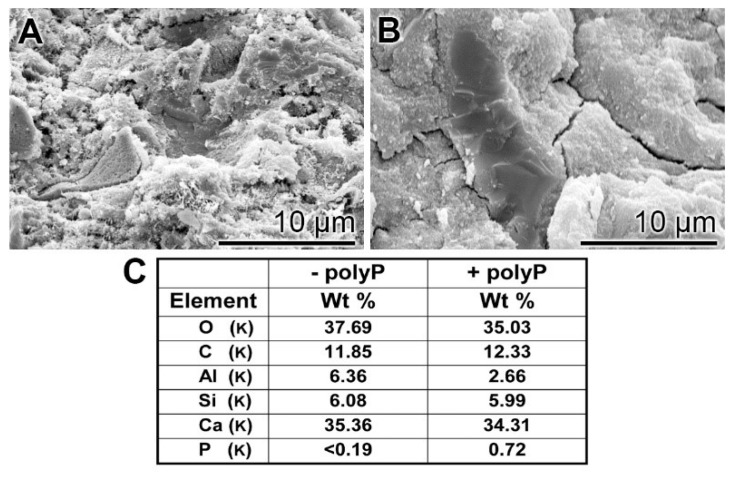
EDX (Energy-dispersive X-ray spectroscopy) analysis of (**A**) hydrated cement “hCEM”, after 3 days, and of (**B**) polyP-containing cement “hCEM•polyP” (maturation time 3 days); SEM analysis was used. The respective spectra were recorded and subjected to a (semi)quantitative evaluation (**C**). The elements O, C, Al, Si, and Ca in the cement paste are listed, as well as P and Ca in “hCEM•polyP”.

EDX (Energy-dispersive X-ray spectroscopy): The presence of polyP in “hCEM•polyP” was demonstrated by EDX analysis and the relative amounts were assessed by (semi)quantitative EDX analysis as well (Figure 3). The samples “hCEM” (Figure 3A) and “hCEM•polyP” (Figure 3B) were subjected to the analysis and the results are given in the table in Figure 3C. It is apparent that the dominant elements in both cement samples are O, C, and Ca, followed by Al and Si. Only in the polyP-containing “hCEM•polyP” sample is P also detected, which amounted to 0.72 weight-%, as expected (Figure 3C). 

**Figure 4:**
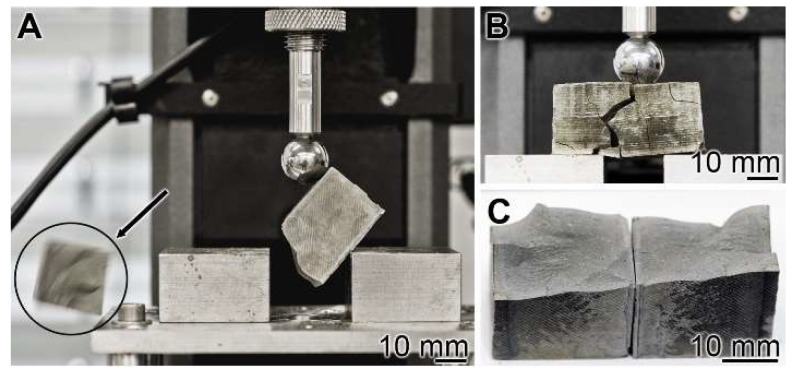
Three-point bending system used for the assessment of the resistance of a cured cement paste specimen. (**A**) The experiments were performed with the system described in Materials and Methods. In the compression region the bursting phase of the cement block is shown (see circle and arrow indicating the flying fragment). (**B**) Bursting of a “hCEM•polyP” sample. (**C**) The corresponding breakthrough surface.

Mechanical toughness: 3-point bend test: The experiments were performed with the 3-point bend system (see Materials and Methods). The cement blocks were mounted, and under increasing load, the materials burst (Figure 4A). A lateral view just before the complete burst shows a main crack resulting in the breakthrough of minor collateral fissures (Figure 4B). The breakthrough surface follows the main crack (Figure 4C).

**Figure 5:**
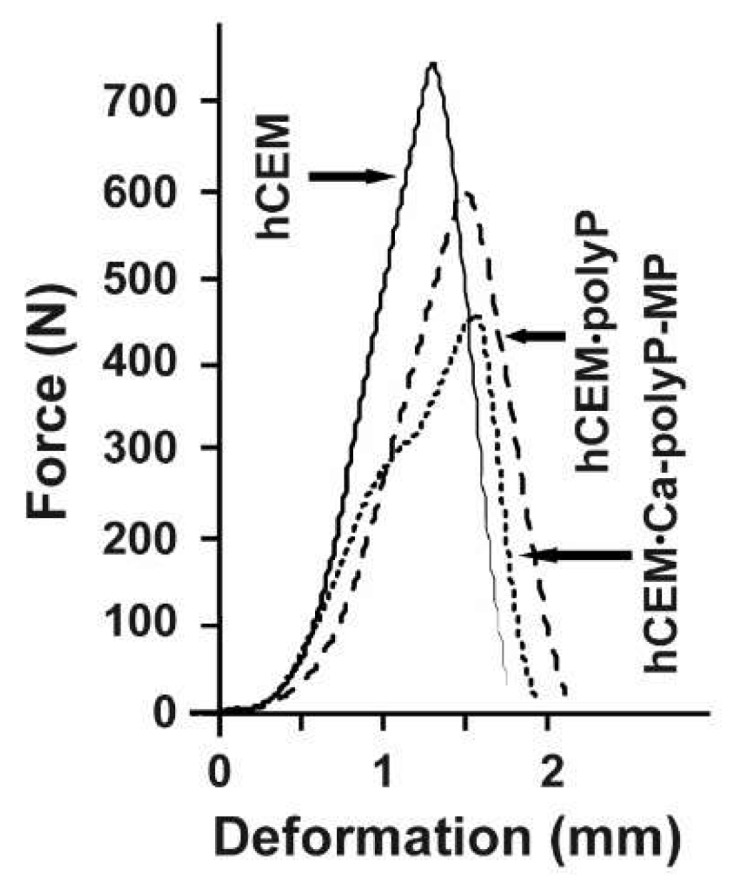
Force-displacement response of different cured cement paste specimens: “hCEM” (continuous line), “hCEM•polyP” (dashed), and “hCEM•Ca-polyP-MP” (dotted).

The respective force/deformation curves of the 3-point bend tests are shown in Figure 5. The control sample, “hCEM”, shows a steep linear increase upon loading. At a maximum loading force of 740 ± 35 N the material fractures, and subsequently breaks through. This process is reflected by the sharp peak within the force/deformation curve, which is followed by a steep decrease. The cement sample, prepared from Na-polyP and CEM I 42.5 R cement “hCEM•polyP”, shows a comparable mechanical behavior. This polyP-containing cement sample shows a reduced mechanical stability, as well as a slightly higher deformation if compared to the control. The maximum loading force was 592 ± 29 N, while the overall force/deformation curve has a similar shape as the one obtained from “hCEM”. In contrast, the load/displacement curve obtained from measurements of the “hCEM•Ca-polyP-MP” shows a biphasic course, which is indicative for a sequential material fatigue. Upon loading, a steep increase comparable to the cement control was recorded. At a load of approximately 300 N a step occurs, which is followed again by a steep linear phase until the maximum load of 450 ± 28 N is reached; then the material breaks. This overall shape of the curve also indicates the occurrence of small intermediate cracks and damages within the material prior to bursting of the sample. The “Cem•Ca-polyP-Emu” samples showed a very similar deformation curve with a maximum load of 480 ± 32 N (material intended for publication).

### 2.2. Old Cement Sample and Surrounding Soil Bacteria Used for PolyP Extraction

**Figure 6:**
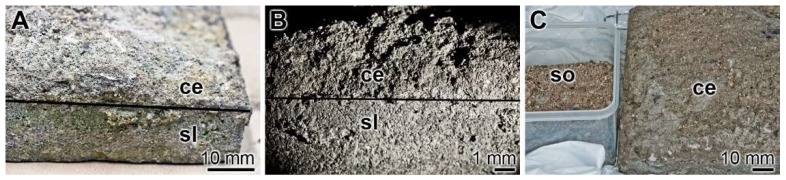
Old cement/concrete sample and the surrounding soil. An approximately 120-year-old sample excavated at the Undenheim-Köngernheim railway station (Mainz; Germany) was used for the study. (**A**,**B**) The cementitious material (ce) was collected at the surface site of a stone slab (sl) facing the subsoil. (**C**) From the cementitious material (ce) splinters were chipped off and bacterial polyP was isolated from the soil (so).

For comparative reasons old cement/concrete specimens that were in the soil for over 100 years and certainly filled with bacteria were collected. Splinters were removed from the cement region directly facing the soil (Figure 6).

The surrounding soil was used for isolation and subsequent cultivation/growth of the bacteria. After cultivation the bacteria were collected by centrifugation and used for isolation of polyP.

### 2.3. Surface Reactivity of the Cement Samples with PolyP

The data from the previous series of experiments indicates that the hydrated cement material, “hCEM”, supplemented with Na-polyP, “hCEM•polyP”, reached almost the same strength if compared with “hCEM”, while addition of “Ca-polyP-MP” in “hCEM•Ca-polyP-MP” lowered the hardness of the material formed. This result is taken as a first indication that Na-polyP undergoes a reaction with the cement, “hCEM”, most likely via exchange of Ca^2+^ originating from the basic cement under formation of Ca-polyP deposits. Consequently, it was wise to study the possible formation of a coacervate on the surface of the cement paste, “hCEM”, in line with our recent observation on coacervate complex formation of polyP [30].

**Figure 7:**
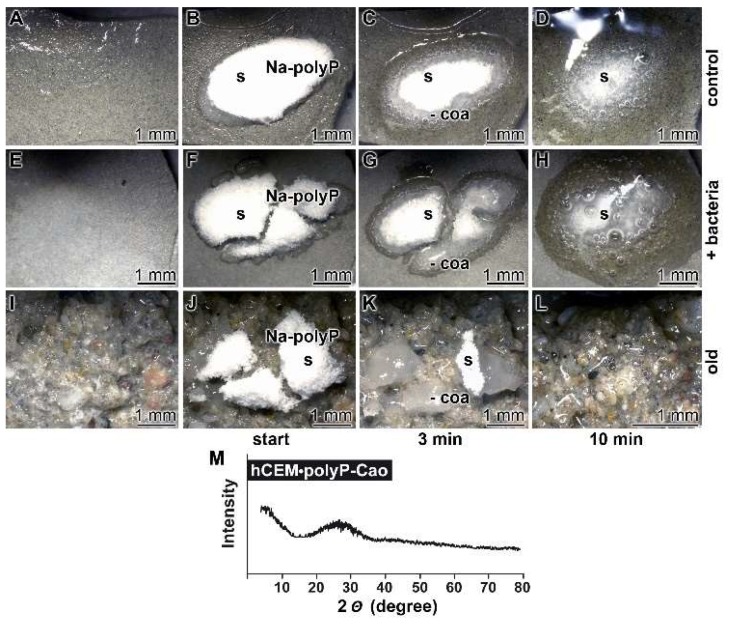
Propensity of the cement surface to support coacervate formation of polyP added as solid Na-polyP, studied with kinetic analysis. Cement blocks were prepared from 42.5 R and water and hardened for 5 d. Half of the heights of the blocks remained deposited into water and the blocks remained in humid chambers. Solid Na-polyP (s) was applied onto each cement block. (**A**–**D**) Coacervate formation onto cement and Na-polyP (control). (**E**–**H**) Placing of Na-polyP onto cement supplemented with the bacteria *E. coli* (+ bacteria), as described under Materials and Methods. (**I**–**L**) Coacervate formation of Na-polyP onto old cement material (old). The time course is marked as follows. In the first panel, no Na-polyP added. The following panels show the remaining solid Na-polyP ((s) white powder (Na-polyP)) immediately after overlay (start) or after 3 min and 10 min, respectively; the coacervate region (coa) is clearly distinguished by the translucent appearance of the material. (**M**) From a control sample the Ca-polyP coacervate was collected for XRD (X-ray diffraction analysis) analysis.

Kinetics of coacervate formation: In this recent report, it is outlined that the conversion of polyP into a coacervate is facilitated and accelerated after having contact with organic molecules or bacteria. Therefore, we soaked the hydrated cement paste “hCEM” either with water or with bacteria (*E. coli*) in medium and exposed those samples in parallel to solid Na-polyP. Eye inspection already showed that the 100 mg of Na-polyP solid material, spread onto the surface of “hCEM”, was rapidly converted into a coacervate (Figure 7A–D). The velocity of transformation was markedly higher if the Na-polyP was applied onto *E. coli*-treated cement (Figure 7E–H) compared to untreated controls (Figure 7A–D). After a period of 10 min, it can be noticed that close to 75% of the initially applied polyP was converted. In a further series, splinters from old cement were collected and overlaid with Na-polyP (Figure 7I–L). In this experiment with the old cement, the transfer of solid Na-polyP to the coacervate is even higher if compared to the prepared samples referred to before.

The nature of the coacervates was determined both by FTIR (material intended for publication; the signals are identical with those shown in Figure 2) and by XRD (X-ray diffraction analysis). A typical X-ray scan reflecting the amorphous character of the coacervate is shown in Figure 7M.

In order to assess the amount of coacervate formed from the 100 mg of Na-polyP, a quantitative conversion study was performed in the absence of cement. Under these conditions, it was determined that 100 mg of Na-polyP form 135 mg of coacervate.

**Figure 8:**
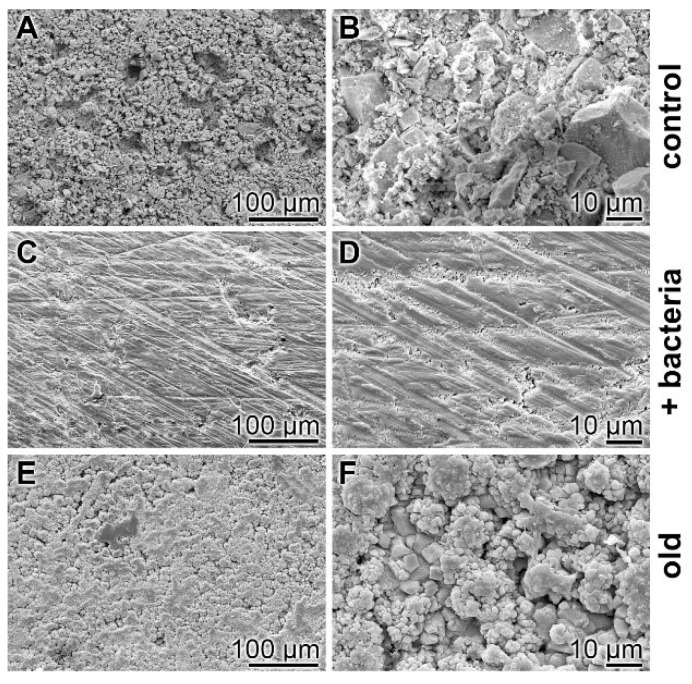
Surface morphology of cement samples (after 5 days maturation) dependent on coacervate formation, analyzed using SEM. (**A**,**B**) Aspect of the control surface of “hCEM” not treated with Na-polyP. (**C**,**D**) The “hCEM” sample was soaked with bacteria and then overlaid with Na-polyP for 10 min to allow coacervate formation. Subsequently, the samples were thoroughly rinsed with water and dried. (**E**,**F**) A similarly sized old cement sample was processed in the same manner with Na-polyP.

Surface characterization—SEM analysis: The surfaces of the cement samples, processed by coacervate formation, were analyzed by SEM (Figure 8). If the non-treated hydrated cement sample, “hCEM”, is inspected as a control, the relatively rippled morphology becomes overt (Figure 8A,B). However, if the “hCEM” sample treated with bacteria is overlaid with Na-polyP for 10 min and examined by SEM after almost complete coacervate formation, thorough rinsing with water, and drying at 50 °C, the crystalline structured surface seen in the control is replaced by an almost smooth surface area (Figure 8C,D). A similar change is seen if the old cement sample is treated with Na-polyP and processed in the same way. The surface of this sample is decorated with a quite spherical layer of deposits, most likely Ca-carbonate crystals (Figure 8E,F).

**Figure 9:**
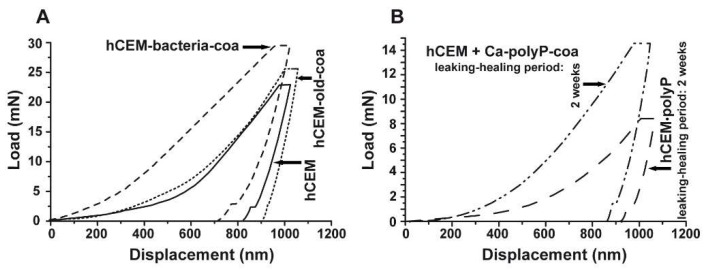
Mechanical properties of the cement surfaces after coacervate formation (**A**,**B**) of the healing products within the cracks. Nanoindentation analysis, as described in Materials and Methods. (**A**) Three samples have been used for the studies; untreated “hCEM”, 5-day-old cement samples treated with bacteria, and then with Na-polyP for coacervate formation (“hCEM-bacteria-coa”), and finally old cement samples processed with polyP coacervate (“hCEM-old-coa”). (**B**) In parallel, the newly formed material within the microcracks was assessed in the same way as the “hCEM•polyP” samples after a 2-week healing period or in “hCEM” cement, into which microcracks were introduced and subsequently sealed with coacervate (“hCEM + Ca-polyP-coa”).

Nanoindentation: The mechanical determinations were performed with the NanoTest Vantage system, as described under Materials and Methods. The respective load/displacement curves for the cement-coacervate material in comparison to the control “hCEM” were determined (Figure 9A). In general, the Na-polyP (coacervate) treatment of the cement surface results in improved mechanical properties compared to the “hCEM” control. This is indicated by a higher load, as well as a steeper slope in the elastic deformation regions of the respective load/displacement curves (Figure 9A). The reduced Young’s modulus of the controls measured 32.59 ± 4.78 GPa (“hCEM”; *n* = 10 different experiments), while the “hCEM” samples were treated with bacteria and then with Na-polyP, which allowed coacervate formation, were stiffer with values of Er = 39.41 ± 10.69 GPa (“hCEM-bacteria-coa”). For the old cement sample also processed by coacervate formation, we measured a reduced Young’s modulus of 35.27 ± 6.53 GPa (“hCEM-old-coa”). Simultaneously, the Martens hardness (MH) values were assessed. For the control “hCEM”, a value of 0.51 ± 0.24 GPa was found, while the two specimens “hCEM-bacteria-coa” and “hCEM-old-coa” show a clearly harder surface with values of MH = 1.41 ± 0.59 GPa for the “hCEM-bacteria-coa” material and MH = 0.98 ± 0.38 GPa for the “hCEM-old” material.

### 2.4. Biomimetic Coacervate Formation onto Cement with Bacterial PolyP

**Figure 10:**
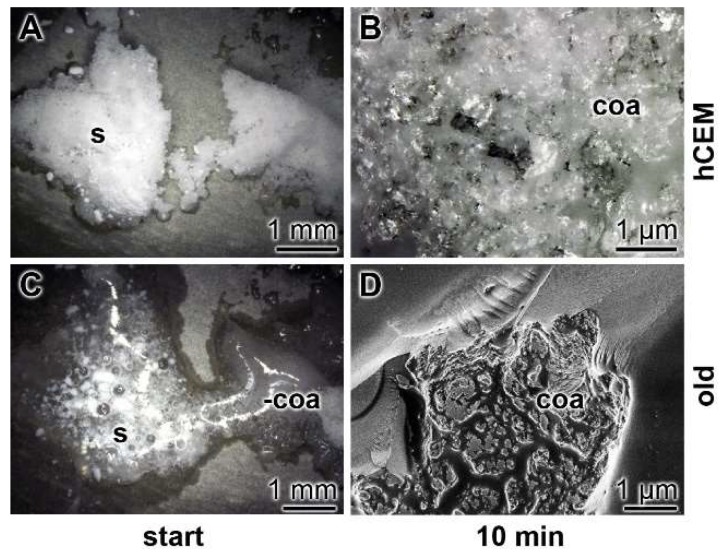
Coacervate formation from natural polyP isolated from bacteria growing next to an old cement sample. Addition of the polymer onto “hCEM” (**A**,**B**). The bacterial solid polyP (s), after addition onto the surface of “hCEM” (5 days old) is still whitish-solid. Application of bacterial polyP onto samples from old cement (**C**,**D**). The polymer immediately undergoes coacervation (coa) on the surface of those samples.

In a parallel series of experiments, polyP was isolated from the soil bacteria, as described under Materials and Methods. Spreading this polymer onto the surface of “hCEM” showed that the bacterial solid polyP already converted to coacervate after 10 min (Figure 10), under otherwise identical conditions, as used for the studies in Figure 7. The Na-polyP material immediately after addition onto the surface of “hCEM” is whitish-crystalline (Figure 10A), while after an incubation of 10 min most of the polymer is converted to the coacervate phase (Figure 10B). The coacervation process onto samples from old cement is much more rapid (Figure 10C,D). Almost immediately, the solid polyP undergoes reaction to coacervation (Figure 10C)

### 2.5. Reactions of CEM I 42.5 R Cement during Exposure to Water and PolyP: FTIR Analyses

**Figure 11:**
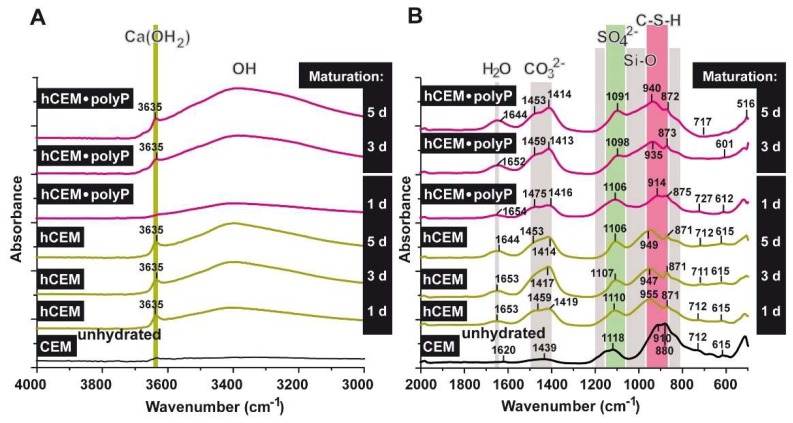
FTIR spectra of unhydrated cement (CEM (unhydrated)), as well as of cement hydrated for 1, 3, 5 days (“hCEM”), and cement supplemented with 1 g Na-polyP/100 g of cement (“hCEM•polyP”). The hydrated cement specimens were matured for 1–5 days, as indicated. The assignments and characterization of the peaks, as well as marked bands, are discussed in the text. The spectra are recorded between (**A**) the wavenumbers 4000 and 3000 cm^−1^ and (**B**) the range between 2000 and 500 cm^−1^.

The FTIR analyses were performed with CEM I 42.5 R before and after water treatment (“CEM”; “hCEM”), as well as with CEM I 42.5 R supplemented with Na-polyP (“hCEM-polyP”), as described under Materials and Methods and shown in Figure 11A,B. All the samples show a complex group of bands between 1650 and 750 cm^−1^ [32,33] and the bands recorded can be attributed to signals, as described in the literature [34,35]. In general, within the range between 800 and 1200 cm^−1^ the stretching vibrations of Si-O (condensed silica) are recorded. The shoulder at ≈1100 cm^−1^ in the profile is indicative for SO_4_^2−^. The asymmetric stretching of CO_3_^2−^ is seen between 1400 and 1500 cm^−1^ [36]. Within 1640 to 1650 cm^−1^ the bending vibrations of molecular H_2_O show up, originating from H-O-H; the vibrations at 2800 (material intended for publication)-3700 cm^−1^ reflect the O-H groups in H_2_O or in other -OH composites (Figure 11).

Comparing the non-processed cement “CEM” with “hCEM”, after addition of water, it becomes obvious that the main band in the profile at ≈880 cm^−1^ shifts to ≈955 cm^−1^, reflecting the Si-O stretching vibrations in the C-S-H (calcium silicate hydrate) gels.

The broad band that is observed in the region 3100–3400 cm^−1^ for hydrated cement is caused by the symmetric and asymmetric stretching (*ν*_1_ and *ν*_3_) of water vibration. The band at ≈1650 cm^−1^ is the deformation mode H-O-H (*ν*_2_) of the adsorbed molecular water. It is important to mention that the (O-H) stretching vibration is clearly observed for hydrated cement after one day as a sharp band at ≈3635 cm^−1^ (stretching of OH in Ca(OH)_2_); this signal becomes stronger with increasing duration of the hydration reaction of the cement due to the formation of calcium hydroxide. The bands of 1439 and ≈880 cm^−1^ are attributed to *ν*_3_, *ν*_2_, and *ν*_4_ CO_3_^2−^, respectively. They also shift to 1459 and 871 cm^−1^ after cement hydration. The presence of CaCO_3_ is the result of the integration of the atmospheric CO_2_ that is absorbed to the system during the hydration of the sample in contact with the air. However, these bands were found in both non-hydrated and hydrated cement. The important feature of the hydrated cements is the displacement of stretching mode Si-O (*ν*_3_) from ≈910 cm^−1^ in the non-hydrated cement to ≈950 cm^−1^ in the hydrated one. This indicates the deposition of the orthosilicate units SiO_4_^2−^ present in tricalcium silicate (C_3_S) and dicalcium silicate (C_2_S) to form calcium silicate hydrate phase (C-S-H). The presence of SO_4_^2−^ ions is reflected by the peak at ≈1118 cm^−1^ (S-O stretching), which is shifted to ≈1110 cm^−1^ after cement hydration. Comparing the band of silicate at 910 cm^−1^ with the polyP-containing cement ones, it is obvious that the shift within the silicate band was not strong after 1 day (≈914 cm^−1^) but increased to 935 cm^−1^ after 3 days. Moreover, the Ca(OH)_2_ band at 3635 cm^−1^ (OH stretching) was very weak in “hCEM-polyP” after one day and became clear and strong after 3 days. Taken together the above results indicate that Na-polyP slightly retards the formation of C-S-H.

### 2.6. Self-Healing of Microcracks in Different Cement Samples

**Figure 12:**
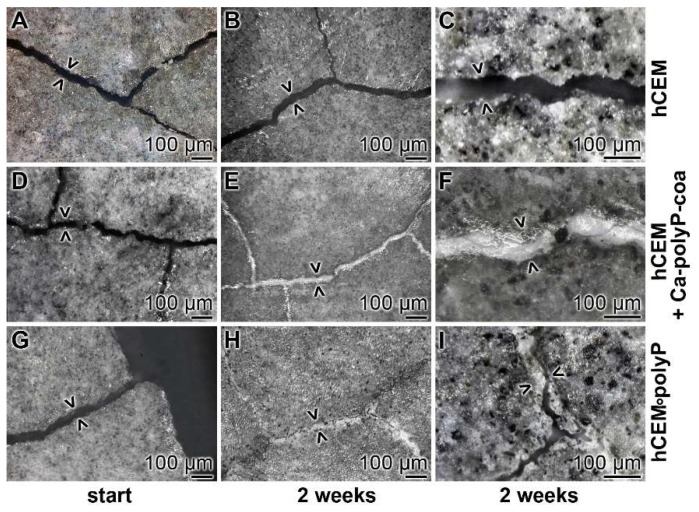
Self-healing of microcracks in polyP-enriched cement. Three series of cement samples with microcracks (some are marked > <) were used in this series: (**A**–**C**) “hCEM”; (**D**–**F**) “hCEM”, into which the microcracks were filled with polyP coacervate (prepared from Na-polyP, CaCl_2_, and water) immediate before the beginning of the experiments (hCEM + Ca-polyP-coa); and (**G**–**I**) “hCEM•polyP”, prepared from CEM with Na-polyP. It is apparent that the cracks in the polyP-free “hCEM“ remained unchanged during the 2 week incubation period, while within the polyP coacervate-treated cracks in “hCEM” (hCEM + Ca-polyP-coa) the deposition of a solid material is seen. Likewise, the deposits seen in “hCEM•polyP” at the end of the incubation period are impressive.

Cement samples “hCEM” and “hCEM•polyP” were prepared, then microcracks (42 ± 20 µm) were induced, and finally the blocks were stored in a humid chamber, as described under Materials and Methods. The crack patterns—formed after thermal gradient/thermal shock cycles—within the cement samples are shown in Figure 12 A,D,G. Immediately after the experiments, or two weeks later, the cracks were inspected by light microscopic observations after drying the samples at 50 °C for 3 days (Figure 12).

Microscopic assessment: In the polyP-free cement sample, “hCEM”, almost none of the microcracks showed any sign of new depositions after 2 weeks (Figure 12A–C). Only very occasionally were small protrusions projected into the cracks. In contrast, the polyP coacervate that was prepared from Na-polyP, CaCl_2_ and water, and rubbed only once at the beginning of the experiments into the microcracks and then processed in the humid chamber showed a homogeneous resealing of the cracks after 2 weeks (Figure 12D–F). Interestingly, for the “hCEM•polyP” specimens that were left in the humid chamber for the 2 weeks incubation period, most of the microcracks were resealed or even partially repaired (Figure 12G–I).

Nanoindentation experiments: The hardness of the newly formed materials within the microcracks of “hCEM•polyP” samples was determined in comparison to the strength of the coacervate that was introduced into the cracks of “hCEM” and remained there for 2 weeks, namely the “hCEM + Ca-polyP-coa” specimens (Figure 9B). The material within the cracks was subjected quantitatively to nanoindentation, as described in the Material and Methods. The results deduced from the corresponding load/displacement curves are depicted in Figure 9B. The material formed in the cracks of the “hCEM + Ca-polyP-coa” samples was determined to have a stiffness of Er: 15.52 ± 6.17 GPa and a Martens hardness of 0.55 ± 0.21 GPa, while the values for the “hCEM + polyP” samples were lower. Values of Er: 9.40 ± 4.89 GPa and MH: 0.23 ± 0.11 GPa have been determined. The superior mechanical properties of the “hCEM + Ca-polyP-coa” samples can also be easily recognized by the shape of the respective curve (Figure 9B), which corresponds to the applied load.

**Figure 13:**
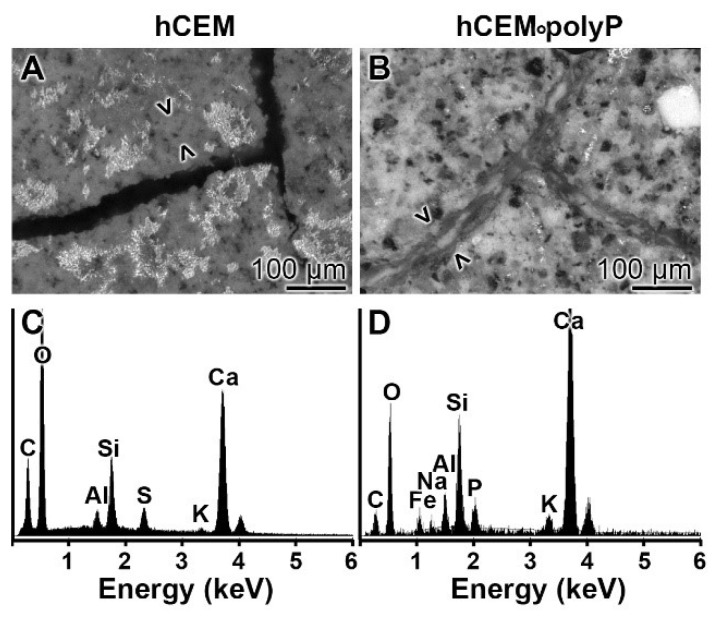
EDX (X-ray diffraction analysis) analysis of (**A**,**C**) the basic material of “hCEM” and of (**B**,**D**) the newly formed material within the cracks of “hCEM•polyP”. (**A**,**B**) SEM inspection and (**C**,**D**) corresponding EDX spectra.

EDX analysis: The chemical nature of the material formed in microcracks of “hCEM•polyP” after an incubation period of 2 weeks was determined by EDX (energy-dispersive X-ray spectroscopy). While in the control cement “hCEM” the dominant elements of polyP-free cement were recorded (O, C, Ca, Al, and Si) on the newly formed bulges within the microcracks (Figure 13A,C), while the material formed on the material during self-healing within microcracks in “hCEM•polyP” also showed a distinct peak for P (Figure 13B,D).

### 2.7. Na-PolyP Addition to Cement—Sand Mixture

Natural quartz sand with a grain size of 0.1–0.4 mm was mixed in a weight ratio of 1:3 cement:sand, as described under Materials and Methods. Those samples termed “hCEM-sand” were used for SEM inspection and hardness assessment. In parallel, the cement-sand material was supplemented with 0.5% Na-polyP (final concentration), named “hCEM-sand•polyP”.

**Figure 14:**
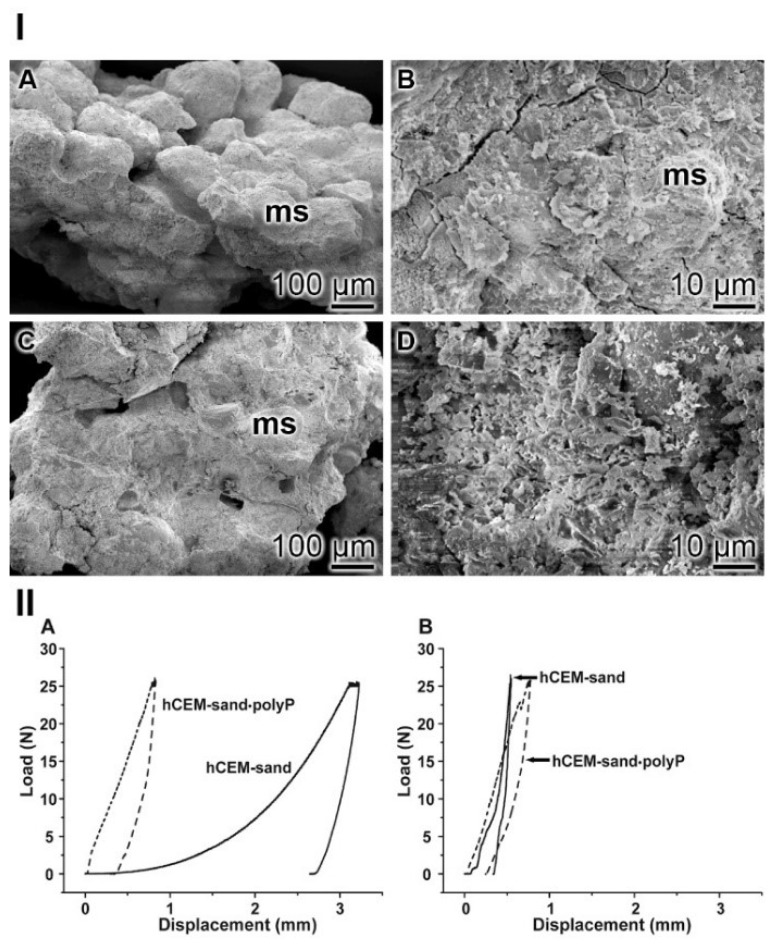
Cement-sand fabricated from CEM I 42.5 R cement and gravel/sand. (**I**) SEM inspection. (**IA**,**B**) The polyP-free cement with micro sand crystals (ms). (**IC**,**D**) In the parallel series polyP-enriched cement was studied. (**II**) Mechanical properties of the cement-sand material. (**IIA**,**B**) In parallel experiments polyP-free “hCEM-sand” (solid lines) and “hCEM-sand•polyP” samples (dashed lines) were measured after (**IIA**) 2 h or (**IIB**) 24 h.

SEM assessment: In the control “hCEM-sand” samples, the cement binder left considerable areas open and did not tightly embed the stone crystals (Figure 14I–A). The polyp-containing cement sample “hCEM-sand•polyP” likewise showed sand stones but the edges were surrounded by the cements (Figure 14I–C). At higher magnification the contours of the stones are distinctly seen (Figure 14I–B), while they are often not apparent in the polyP-enriched cement-sand (Figure 14I–D).

Nanoidentation: The different morphological appearances within the “hCEM-sand” as well as of the “hCEM-sand•polyP” sample are reflected by the load/displacement curves calculated from the compression tests. The recordings from the control sample, when compared to the polyP-containing cement-sand, showed striking differences when analyzed 2 h after mixing. While the control curve “hCEM-sand” follows a long, nonlinear increase after loading until reaching the maximum (25 N), likely to be due to the squeeze out of the unbound water in the sample, the second half of the curve can be described by a steeper slope (Figure 14IIA). At the maximum, a creep phase is pronounced. Furthermore, a distinct displacement of 3.2 mm at maximum load was recorded. Upon unloading the curve shows a steep decrease with only little recovery, which amounts to 19% of the maximum deformation. In contrast, for the polyP-containing “hCEM-sand•polyP” sample the loading curve shows a relatively short and steep linear increase. After reaching the maximum load of 25 N, only a slight creep behavior of the material was recorded. The maximum displacement of the polyP-enriched cement-sand amounted to 0.8 mm. Upon unloading the curve shows a steep decrease with a recovery of 65%, which is more than three times higher compared to the recovery of the “hCEM-sand” control.

As expected, after 1 day of incubation the mechanical properties of the cement samples, in particular those of the cement-control, changed considerably (Figure 14IIB). Interestingly, the two curves then became more similar. The “hCEM-sand” sample showed an almost linear fast and steep increase without any noticeable creep at maximum load (25 N). The total displacement at the end of the loading period amounts to 0.5 mm. Upon unloading, the decrease was likewise fast and steep and resulted in a displacement recovery of 30%. The recording of the polyP-containing “hCEM-sand•polyP” sample showed a similar linear, fast, and steep increase. At a load of 22.5 N, a small peak became apparent. This peak most likely resulted from a minor crack within the sample. After reaching the maximum load (25 N), a short creep phase was observable. At the end of the loading period, the total displacement amounted to 0.8 mm. The subsequent steep unloading phase reflects the recovery of the material, which amounts to almost 67%, a number which matches the one determined for the 2-hour-old cement-sand sample.

## 3. Discussion

Since small invisible or inaccessible cracks are difficult to repair, and over time become severe and reduce the stability of concrete, improvement of the self-healing capacity of cracked concrete is advisable. Basically, the self-healing of concrete and clinker is an old phenomenon, since concrete possesses genuine, natural, autogenous healing properties [37]. This process is based on continuous hydration of the minerals or carbonation of Ca(OH)_2_ resulting in a (limited) healing of cracks, or both. More recently, efforts have been undertaken to incorporate autonomous crack-healing properties [38]. Since 2001 the self-healing capacity of polymer-based materials has become popular [39].

**Figure 15:**
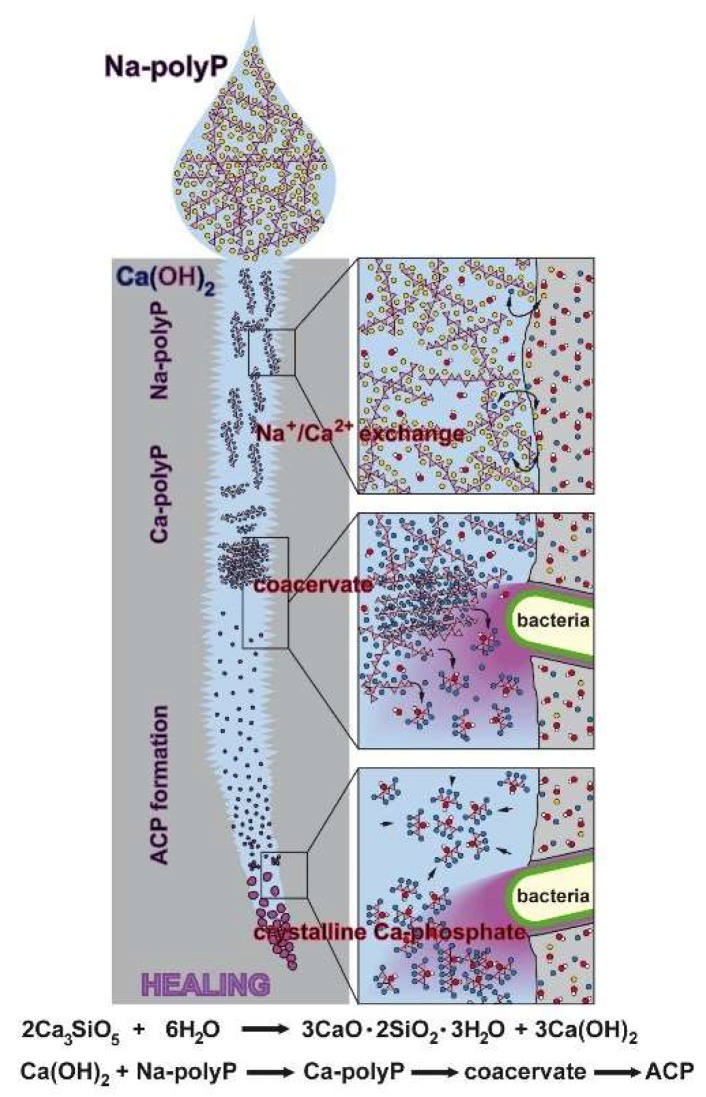
Proposed reaction chain of polyP in microcracks of cement. Initially the soluble Na-polyP is converted to Ca-polyP by the Ca^2+^ ions resealing in dynamic processing and maturation steps of the microcracks in the cement. After this Na^+^/Ca^2+^ exchange the two bacteria-driven reactions follow, namely the enzymatic hydrolysis of polyP to monomeric phosphate and the conversion to the amorphous Ca-phosphate (ACP). Finally, the conversion to crystallinic Ca-phosphate takes place. This process is purely thermodynamically driven, without the application of an enzyme.

The self-healing of cement-based materials can be classified into three groups: intrinsic healing; autonomic, capsule-based healing; and vascular healing [37]. In our approach we focused on the autonomic mechanisms by adding polyP to the cement material prior to concrete fabrication or later by lubrication into the cracks (microcracks). This polymer, added as Na-polyP, is predestined for such a healing approach, since it comprises the two relevant properties required to readily undergo Na^+^/Ca^2+^ exchange and secondly to go through the phase of coacervation (Figure 15); this phase of polyP is characterized by a high water content linked with a high degree of molecular mobility. The exchange by Na^+^/Ca^2+^ triggers the overall dynamic chemical processes in the cement and is also involved in corrosion resistance [40]. In addition, this process facilitates and accelerates the transitory coacervate formation [30], starting with water-soluble polyp, which is transformed in the presence of Ca^2+^ into the water-rich and likewise dynamic coacervate, prior to transition to the crystalline Ca-phosphate phase. Importantly, in this process intrinsic bacteria fulfill important functions, e.g., by hydrolyzing the polymer to monomeric phosphate via the bacterial enzyme alkaline phosphatase [41], and secondly by promoting the transition of polyP to crystalline Ca-phosphate [42].

Analytical studies revealed that, essentially as expected, addition of polyP (*i*) increases the phosphate content of the cement (EDX measurement) and (*ii*) shifts the bending vibration signals especially for the P-O-P stretching (FTIR). Furthermore, morphology analysis using SEM showed that the cement deposits became more compact after addition of Na-polyP, also reflected by an almost similar deformation resistance (3-point bending assay). Important is the finding that the surface of the polyP-supplemented cement still retains the property to undergo Na^+^/Ca^2+^ exchange after addition of Na-polyP followed by coacervation. This finding shows the potency of Na-polyP in forming a tight interaction with the cement minerals, driven by their Ca^2+^ ingredient. This process results not only in a tight interaction of the cement but also supports and prolongs the dynamic property of the cement. The polyP coacervates formed on the cement showed the characteristics of the control coacervate preparation, not in contact with cement, to be amorphous (XRD) and matching with the vibration signals (FTIR).

In order to elucidate if polyP, encapsulated into microparticles or into microspheres, increases the stability of the cement, those particles were prepared by co-precipitation with Ca^2+^ or by trapping into PVA (polyvinylalkohol) and were then added to the cement. The experiments revealed no additional recognizable improvement in the mechanical stability (deformation/force curve). Therefore, this line of development was not followed further in the undertaken experiments.

FTIR analyses [33,34] revealed that in polyP-containing cement the formation of the signal for Ca(OH)_2_ decreased, followed by CaCO_3_ formation. Together with the Si-based minerals the connected calcium silicate hydrate phase (C-S-H) became modulated, showing that Na-polyP retards the formation of C-S-H, a finding that might have an impact for future applications of polyP supplementation in cement for exploitation in practice.

Interesting is the finding that the coacervate formation onto 120-year-old cement showed substantially faster kinetics compared to a recent cement sample. In order to prove the hypothesis that the intrinsic bacteria in the old cement are responsible for this change, one series of experiments was performed with newly prepared cement samples that were penetrated with bacteria. An increased coacervation was also observed in those samples. Parallel to these studies, the morphology and texture of the cement samples were determined by SEM. It was observed that the surface of cement samples incubated with bacteria and subsequently treated with polyP to allow coacervate formation showed a smoother texture compared to the untreated cement controls. The surface of the old cement sample treated with polyP coacervation also showed a lower crystallinic texture. Likewise, the finding that the hardness values of the cement samples infiltrated with bacteria, and also those of old cement (both of them being subsequently treated with polyP to allow coacervate formation), are superior compared to the controls was striking.

These results can already be taken as a further strong hint that bacteria can accelerate coacervate formation. As direct proof that the polyP component of bacteria is crucially involved in the observed accelerating effect, the polyP polymer was isolated from soil bacteria. Those natural polyP samples showed the same property of coacervation onto cement as the synthetically produced polymer. Based on this battery of evidence we postulate that the ability of soil bacteria to support self-healing of cement/concrete-based materials is not only due to the release of metabolic CO_2_ linked to subsequent Ca-carbonate production from intrinsic Ca(OH)_2_ [43] but also due to the polyP-driven mineralization of the cement. Such a view could qualify the polyP-driven cement hardening, and especially the increased self-healing capacity of the polyP-enriched cement, not only as a bioinspired but also as a biomimetic process.

Following this bio-inspired concept of a bacteria-based, self-healing, natural engineering mechanism, the potency of polyP was determined, added either intrinsically, together with the cement, or extrinsically, perhaps related to the vascular based self-healing materials sequestration. Microcracks were introduced into the cement. While after 2 weeks in the polyP-free material no traces of sequestered material could be identified in the cracks, this was strikingly different in cement supplemented with Na-polyP. The cracks in those cement samples comprised voluminous sequestered material identified as minerals, mainly composed of Ca, Si, P, Al, and O (shown with EDX analysis). From this data it can be deduced that the material released into the crack fractures contain important ground elements of cement like Si and Al, in addition to phosphate. This finding let us to suggest that—most likely driven by the polyP-based dynamic chemical conversion processes—cement constituents are released into the fissures. The introduction of coacervate into the cracks resulted in perfect sealing after 2 weeks. Mechanical measurements revealed that the mechanical and hardness properties of the sequestered material was ≈35% for the intrinsic, polyP-cement released material, and ≈65% for the extrinsically applied polyP coacervate, compared to the surrounding material. In our view, this finding might qualify polyP as a valuable additive to cement to increase the self-healing capacity.

In a final series of experiments it was demonstrated that polyP added in a final concentration of 0.5% to a cement/sand mixture substantially shortened the hardening period immediately after preparation of the cement with water. After 24 h the hardness of the material—both polyP-free and polyP-containing cement/sand—was very much similar. This difference can surely be modified by changing both the water and the polyP content in the material. A rapidly hardening Portland cement-sand blend is needed for various applications, such as for insulating foam or cast-frame construction [44]. Furthermore, the rate of hardening of Portland cement-sand can be controlled by addition of gypsum [45].

## 4. Materials and Methods

### 4.1. Materials

The Portland cement, CEM I 42.5 R cement, was a gift of the Heidelberg Cement AG (Dr. Ulrich Schneider, Mainz; Germany). The batch used (LN 43308/001) contained 63.6% Ca_3_SiO_5_ (weight-%), 1.6% α-Ca_2_SiO_4_, 4.3% β-Ca_2_SiO_4_, 6.4% aluminate (Ca_3_Al_2_O_6_) (cubic and orthorhombic), 12.6% ferrit (Ca_2_(Al, Fe)_2_O_5_], 1.9% K_2_SO_4_, 0.5% MgO, 0.3% CaO, 1.5% Ca(OH)_2_, 2.6% CaSO_4_, 2.7% CaSO_4_•0.5 H_2_O, 0.8% CaCO_3_, 0.4% SiO_2_, and 0.6% CaMg(CO_3_)_2_). Na-polyphosphate (Na-polyP), with an average chain length of 40 phosphate units, was obtained from Chemische Fabrik Budenheim (Budenheim; Germany).

### 4.2. Preparation of the Ca-polyP Microparticles

The calcium polyP microparticles, termed “Ca-polyP-MP”, were prepared from Na-polyP as described [29]. A 2:1 weight ratio between CaCl_2_ and Na-polyP was chosen. In brief, 2.8 g of CaCl_2_•2H_2_O (#223506, Sigma-Aldrich, Taufkirchen; Germany) were dissolved in 25 mL distilled water and added to 1 g of Na-polyP in 25 mL distilled water at room temperature at a pH of 10. After stirring microparticles (MP) were formed, which were collected by filtration, washed with ethanol, and dried [46]. The microparticles, “Ca-polyP-MP”, had a size of ≈160–250 nm in diameter.

### 4.3. Preparation of PolyP Spheres

The emulsion technique was applied, as outlined in a previous study [47]. In brief, 10 g of poly(vinyl alcohol) (PVA) (M_w_ 146,000–186,000; #363065, Sigma-Aldrich) were dissolved in 100 mL of distilled water at 90 °C while stirring for 3 h. This sample was added to 200 mL of paraffin oil (#18512, Sigma), supplemented with 10% (*wt*/*wt*) Tween-80 (#P4780, Sigma). Then, 10 mL of a CaCl_2_ solution (stock solution of 1 g CaCl_2_/10 mL water) were simultaneously dropped in parallel with 10 mL of a Na-polyP solution (stock solution of 0.2 g/10 mL water) into the assay. After stirring, the particles were obtained by sedimentation. The spheres formed were freeze-dried at −80 °C and termed “Ca-polyP-Emu”. While the polyP microparticles have a size of ≈200 nm, the spheres reach a diameter of up to 3 µm, with an average of 850 ± 170 nm (scanning electron microscopic analysis). The PVA “shells” have dimensions of < 50 nm.

### 4.4. Preparation of the Cement Samples

“CEM”/“hCEM”: The unhydrated cement was termed “CEM”. The control pastes were prepared by mixing of 100 g of CEM I 42.5 R cement with 38 g of distilled water. The resulting hydrated cement was termed “hCEM”. During this procedure (30 min) the temperature increased slightly to ≈28 °C.

“hCEM•polyP”: The same ratio of cement:water was used for Na-polyP supplementation. If not mentioned otherwise, 100 g of CEM I 42.5 R were supplemented with 1 g of Na-polyP and reacted with 38 g of water; the resulting paste was termed “hCEM•polyP”. In some additional studies, the polyP amount was increased to 3 g or 5 g, respectively.

“Ca-polyP-MP”/“hCEM•Ca-polyP-MP” and “Ca-polyP-Emu”/“hCEM•Ca-polyP-Emu”: If “Ca-polyP-MP” was added, 3 g of the microparticles (“Ca-polyP-MP”) were mixed with 100 g of CEM I 42.5 R and 45 g water to obtain “hCEM•Ca-polyP-MP”. In the case of the cement samples containing “Ca-polyP-Emu”, 100 g of cement were supplemented with 3 g of “Ca-polyP-Emu” and 38 g of water (“hCEM•Ca-polyP-Emu”).

The pH in the cement after addition of water remained at ≈12, while in the experiments with the polyP supplement the pH increased slightly and reached values up to 13.

If not mentioned otherwise, the different samples were allowed to harden in 5 cm × 2.5 cm × 2.5 cm plastic molds for 5 days prior to further analysis.

### 4.5. Cement Samples for Microcrack Formation and Self-Healing Analysis

The “hCEM” and “hCEM•polyP” samples were prepared following the standard procedure. Cement blocks measuring 5 cm × 2.5 cm × 2.5 cm were maturated for 10 days. Then, microcracks were introduced into the cement samples by a shock freeze–warming cycle. For this, the cement samples (used 10 day after preparation of the blocks) were submersed into liquid nitrogen (−196 °C) for 5 h, followed by one heating cycle to 80 °C. The diameters of the resulting cracks ranged between 15 and 50 µm, with an average of 42 ± 20 µm (25 determinations). One day prior to the experiment, the blocks were submersed in water reaching 3 mm below the top edge of the blocks. The specimens were stored in a humid chamber. In one series of experiments the cracks were filled with the coacervate, “hCEM + Ca-polyP-coa”, and remained in the chamber for 2 weeks before analysis of the hardness.

### 4.6. Old Cement and Surrounding Gravel/Sand

An old cement sample (about 120 years old) was collected together with the surrounding gravel/sand at the train station of Undenheim-Köngernheim, Mainz, Germany. From this sample splinters were removed mechanically.

### 4.7. Coacervate Formation onto Surface of Hardened Cement Paste

Cement paste was prepared following the standard procedure (mixing of 38 g CEM I 42.5 R with 100 g of water), filled into plastic forms, and allowed to harden for 5 day. In this series of experiments the height of the cubes that formed was 6 mm, which were laid onto filter paper in a humid chamber that was filled with water until reaching a level of 3 mm. In one series the cubes were processed without further treatment, or in the second one they were incubated with *Escherichia coli* (K-12 strain) at a density (OD_600_) of 0.45 for 30 min. Then, 100 mg of Na-polyP (as dry salt) were layered onto the non-submersed surface and eye-inspected for coacervate conversion. A third sample was chosen from the old cement material, having a similar size, and treated in the same way with polyP. The samples remained in the humid chamber for up to 10 min. Continuous light microscopic recording was performed.

As reference material, the coacervate was prepared from Na-polyP, CaCl_2_, and water as described before [30].

### 4.8. Mixing of Cement with Sand

Natural furnace-dried quartz sand with a grain size of 0.1–0.4 mm was obtained from Min2C GmbH (Melk; Austria; cat. number # MQS0104TNA-V1). In the controls, termed “hCEM-sand”, cement and quartz sand were mixed at a cement/sand ratio of 1:3 and then supplemented with water at a water/cement ratio of 1:2. Subsequently, the slurry was poured into the standard molds (5 cm × 2.5 cm × 2.5 cm). The samples were analyzed after 2 h of incubation and after 24 h, respectively.

For the experiments shown here, Na-polyP was dissolved (0.1 g/mL) in water and then added to the dry cement-sand mixture at the 1:3 ratio until a final concentration of 0.5% Na-polyP (with respect to cement) was reached. This “hCEM-sand•polyP” material was further processed.

### 4.9. Isolation of Microbial PolyP

Soil bacteria were collected from the humic loess soil at Undenheim-Köngernheim [48] and cultivated as described [49,50]. In brief, soil samples of ≈10 mg were taken and suspended in 1.0 mL of 0.85% (*w*/*v*) NaCI solution. After vortexing the cells were separated from the soil particles and diluted in M9 minimal medium (Sigma). Then, the samples were diluted in Difco Nutrient Broth (Sigma). After reaching an OD_600_ the bacteria were collected by centrifugation and used for polyP extraction, following the described procedure [51]. Samples were successively extracted by applying a procedure that avoids hydrolysis of long-chain polyP [52]. Protein was removed from the extracts with phenol/chloroform (1:1 (*v*/*v*)), followed by three successive extractions with chloroform. The polyP content was determined spectrophotometrically using a Beckman DU-64 spectrophotometer [51,53]. The resulting polyP yield was 70 µg/g bacteria (wet weight), following the established determination method [52].

### 4.10. Fourier Transformed Infrared Spectroscopy

The analysis by Fourier transformed infrared spectroscopy (FTIR) was performed with an attenuated total reflectance-FTIR spectroscope/Varian IR spectrometer (Agilent, Santa Clara, CA, USA). The ground powder was analyzed.

### 4.11. EDX Analysis

The experiments were performed with an EDAX Genesis EDX (energy dispersive X-ray analysis—Genesis—energy-dispersive X-ray spectroscopy) System attached to a scanning electron microscope (Nova 600 Nanolab, FEI, Eindhoven, The Netherlands). The analyses were operated at 10 kV with a collection time of 30–45 s. Areas of 10 µm^2^ were analyzed. The system was calibrated with standard samples, allowing measurements with an error of approximately 10% [54].

### 4.12. Microscopic Analysis

For the SEM (scanning electron microscope) studies, an ESEM (environmental scanning electron microscope) XL-30 machine (Philips, Eindhoven, The Netherlands) was applied [55]. Light microscopic inspection was performed with a VHX-600 Digital Microscope (Keyence, Neu-Isenburg, Germany) equipped with a VH-Z25 zoom lens.

### 4.13. Mechanical Toughness: 3-Point Bend Testing

The fracture toughness of the hardened cement paste materials was determined by means of a 3-point bend system (5940 Series system; Instron, Norwood, MA, USA), following the procedure described previously [56]. For the measurements, we used the standardized spherical head (diameter of 18 mm) and defined cement paste samples (50 mm (length) × 25 mm (width) × 25 mm (height)). The samples were placed on top of two prismatic bars, spacing 40 mm to each other. The load was applied with an acceleration of 5 mm/min to the center of the sample body. All samples measured were loaded until breakthrough while continuously recording force, deformation, as well as process time.

### 4.14. Local Mechanical Properties—Nanoindentation

The determinations of the mechanical properties of the surfaces of the samples, like the hardness as well as the local reduced Young’s modulus, were performed using the nanoindentation NanoTest Vantage System (Micro Materials Ltd., Wrexham, UK), mounted with a Berkovich diamond indenter. For each sample 30 independent measurements with 30 µm spacing in between the indentation sites were performed. We used a depth controlled approach with a fixed indentation depth of 1 µm. The loading and unloading rates were set to 1 mN/s, respectively. After reaching the respective indentation depth, the force was kept there for 30 s before the subsequent unloading phase was started. All measurements were performed at 25 °C. The Martens hardness as well as the Young’s modulus were calculated following the described procedure [57]. All calculations were performed with the NanoTest Platform Four V.40.08 software package (Wrexham LL13 7YL, UK).

The hardening of the cement paste materials with or without Na-polyP was assessed by compression testing using a “MultiTest 2.5-xt Force Testing System” equipped with a 100 N Load Cell unit (Mecmesin Ltd., Slinfold, UK). The cement paste samples were loaded in the longitudinal direction with a loading speed of 5 mm/min using a 14 mm load-button. A load of 25 N was selected for the samples, which was kept constant for 60 s. Subsequently, an unloading period (0 N) followed for 300 s. The force-displacement-time data were continuously recorded at a frequency of 50 Hz using the Emperor XT Force software (Mecmesin Ltd.).

## 5. Conclusions

In the last few decades of research on self-healing additives to cement a wide variety of self-healing concepts has been developed. Great emphasis has been put on strategies based on new healing chemistries, considering stability, higher reactivity, and faster kinetics. In the present study, we focused on the application of polyP, added as water-soluble Na-polyP, to Portland cement, and its effects on the self-healing properties of this binder. Our research strategy was driven by the concepts of biomimetics and bioinspiration. Conclusive data are presented indicating that soil bacteria have the potency to initiate and accelerate self-healing. The natural polymer polyP, an important physiological component of bacteria, can either be intrinsically extruded or extrinsically applied into microcracks, where it subsequently seals the fissures according to an evidence-based sequence, which is schematically outlined in Figure 15. Initially, the highly water-soluble Na-polyP undergoes Na^+^/Ca^2+^ exchange resulting in formation of a coacervate, a dynamic water-rich Ca-polyP phase. In parallel or sequentially the polyP is enzymatically hydrolyzed to monomeric phosphate, which serves as a component for the amorphous Ca-phosphate precipitation. This step might involve bacteria. It should be stressed here that the amorphous phase of Ca-phosphate has been shown to be stabilized by addition of small amounts of polyP [58]. The final phase, the transformation of amorphous Ca-phosphate to the crystalline phase, is apparently again mediated by bacteria. In turn, the self-healing process of polyP in the concrete materials and deposits can be subdivided into the following phases: (1) Transformation of polyP into the coacervate phase due to a shift in the pH. While initially, after being in contact with water, concrete has an alkaline pH, this value decreases during exposure to air (CO_2_) to a more acidic value. (2) During this pH shift polyP undergoes coacervation [30]. In this phase, the polymer readily encompasses the crystalline grains. (3) The surrounding bacteria release alkaline phosphatase and transform the polymer to inorganic phosphate units. (4) These anions bind to Ca^2+^ under formation of crystalline Ca-phosphate deposits. Overall, these processes are comparably slow and require water as well as CO_2_ from air to proceed. We assess the data presented as a further approach towards a commercial, hopefully successful, self-healing concept that is beneficial not only for concrete defect restoration but also for other applications, perhaps in biomedicine.
